# Screening of Hub Genes Associated with Pulmonary Arterial Hypertension by Integrated Bioinformatic Analysis

**DOI:** 10.1155/2021/6626094

**Published:** 2021-03-22

**Authors:** Yu Zeng, Nanhong Li, Zhenzhen Zheng, Riken Chen, Min Peng, Wang Liu, Jinru Zhu, Mingqing Zeng, Junfen Cheng, Cheng Hong

**Affiliations:** ^1^Department of Respiration, The Second Affiliated Hospital of Guangdong Medical University, Zhanjiang, Guangdong, China; ^2^Institute of Nephrology, Affiliated Hospital of Guangdong Medical University, Zhanjiang, Guangdong, China; ^3^China State Key Laboratory of Respiratory Disease, National Clinical Research Center for Respiratory Disease, The First Affiliated Hospital of Guangzhou Medical University, Guangzhou, Guangdong, China; ^4^First Clinical School of Medicine, Guangdong Medical University, Zhanjiang, Guangdong, China

## Abstract

**Background:**

Pulmonary arterial hypertension (PAH) is a disease or pathophysiological syndrome which has a low survival rate with abnormally elevated pulmonary artery pressure caused by known or unknown reasons. In addition, the pathogenesis of PAH is not fully understood. Therefore, it has become an urgent matter to search for clinical molecular markers of PAH, study the pathogenesis of PAH, and contribute to the development of new science-based PAH diagnosis and targeted treatment methods.

**Methods:**

In this study, the Gene Expression Omnibus (GEO) database was used to downloaded a microarray dataset about PAH, and the differentially expressed genes (DEGs) between PAH and normal control were screened out. Moreover, we performed the functional enrichment analyses and protein-protein interaction (PPI) network analyses of the DEGs. In addition, the prediction of miRNA and transcriptional factor (TF) of hub genes and construction miRNA-TF-hub gene network were performed. Besides, the ROC curve was used to evaluate the diagnostic value of hub genes. Finally, the potential drug targets for the 5 identified hub genes were screened out.

**Results:**

69 DEGs were identified between PAH samples and normal samples. GO and KEGG pathway analyses revealed that these DEGs were mostly enriched in the inflammatory response and cytokine-cytokine receptor interaction, respectively. The miRNA-hub genes network was conducted subsequently with 131 miRNAs, 7 TFs, and 5 hub genes (CCL5, CXCL12, VCAM1, CXCR1, and SPP1) which screened out via constructing the PPI network. 17 drugs interacted with 5 hub genes were identified.

**Conclusions:**

Through bioinformatic analysis of microarray data sets, 5 hub genes (CCL5, CXCL12, VCAM1, CXCR1, and SPP1) were identified from DEGs between control samples and PAH samples. Studies showed that the five hub genes might play an important role in the development of PAH. These 5 hub genes might be potential biomarkers for diagnosis or targets for the treatment of PAH. In addition, our work also indicated that paying more attention on studies based on these 5 hub genes might help to understand the molecular mechanism of the development of PAH.

## 1. Background

Pulmonary arterial hypertension (PAH) is a disease or pathophysiological syndrome of abnormally elevated pulmonary artery pressure caused by known or unknown reasons [1, 2]. PAH not only can be caused by the pathological changes of pulmonary vessels themselves but also be secondary to other heart, lung, or systemic diseases [3]. Pulmonary circulatory disturbance and high right heart load are the main characteristics of PAH [3]. Severe PAH can lead to right heart failure and even death. It is reported that the PAH prevalence rate is 15-60 cases/million people/year, and the incidence rate is 5-10 cases/million people/year [4]. In the past few decades, great progress has been made in understanding the basic pathobiology of PAHs and their underlying history, prognostic biomarkers, and treatment options; however, the mortality from PAH remains high. Recently, the US-REVEAL registry displayed a low 5-year survival rate of 61.2% in patients with newly diagnosed PAH [5]. Therefore, it has become an urgent matter to search for clinical molecular markers of PAH, study the pathogenesis of PAH, and contribute to the development of new science-based PAH diagnosis and targeted treatment methods.

Bioinformatic analysis is a tool that can be used to discover potential molecular markers in the pathology of disease by analyzing the differential gene expression between patients and healthy controls [6]. At present, the in-depth study of transcriptome data through bioinformatic analysis provides a new reference for finding new diagnostic molecular markers, prognostic monitoring markers, and therapeutic targets [7, 8]. The primary diagnostic and evaluation method for pulmonary hypertension is invasive right heart catheterization, and the primary therapeutic agent is difficult to achieve a satisfactory therapeutic outcome due to its systemic effect on blood vessels. Therefore, we intend to use the information of PAH patients in the GEO database for bioinformatic analysis to find diagnostic markers and target genes for treatment, so as to reduce the harm caused by invasive diagnostic techniques and reduce the side effects caused by nonspecific treatments. We hypothesized that some genes or proteins found through bioinformatics could contribute to the diagnosis and treatment of PAH more specifically and help for molecular mechanism of PAH.

Through gene expression analysis by chip technology, more data of PAH expression profile are revealed, which is helpful for comprehensive basic research and understanding of biological function of differentially expressed genes (DEGs) of PAH. In this study, a microarray dataset about PAH was downloaded from the Gene Expression Omnibus (GEO) database, and the DEGs between PAH samples and normal samples were identified. Moreover, we performed the functional enrichment analyses including Gene Ontology (GO) and Kyoto Encyclopedia of Genes and Genomes (KEGG) enrichment analyses and protein-protein interaction (PPI) network analyses of the DEGs. In addition, the prediction of microRNA (miRNA) of hub genes and construction of the miRNA-hub gene network were performed. This study is aimed at exploring the hub genes related to PAH by bioinformatic analysis.

## 2. Materials and Methods

### 2.1. Microarray Data Analysis

**Figure 1:**
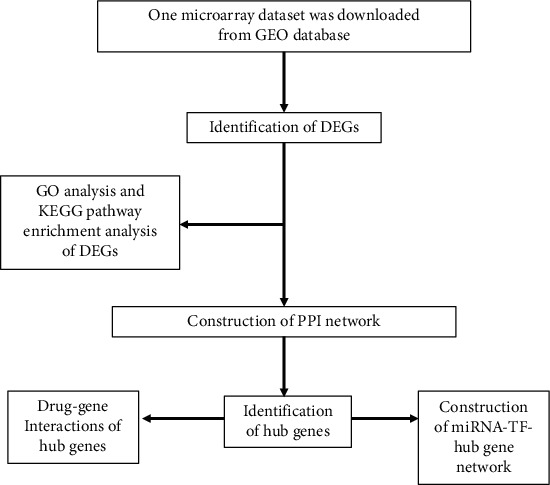
The workflow of this study.

The National Center for Biotechnology Information Gene Expression Omnibus (NCBI-GEO) (https://www.ncbi.nlm.nih.gov/geo/) is a public database created in the year 2000 [9, 10]. It contains the transcriptome data of microarray chips that were submitted by all kinds of institutions around the world. One mRNA expression profiling (GSE117261) was downloaded from the GEO database for further analysis, whose data has been normalized and log2 transformed. The GSE117261 contained gene expression information of 58 PAH lung tissue samples and 25 normal samples. The demo data of the samples in GSE117261 was shown in Table S1. These microarray data were executed with the help of GPL6244 [HuGene-1_0-st] Affymetrix Human Gene 1.0 ST Array [transcript (gene) version]. The detail of GSE117261 was shown in Table 1. To make this article better understand, the workflow of this study was shown in Figure 1.

### 2.2. Data Processing

After GSE117261 was downloaded, probe identification numbers were transformed into gene symbols in *R* software (version 3.6.3 https://www.r-project.org) for further analyses, respectively [11]. The annotation library hugene10sttranscriptcluster.db was used to performed the probesets mapping to their respective gene symbol identifier, and probesets annotated to the same gene symbol identifier were aggregated according to their mean value [12, 13]. In the GSE117261 dataset, the “limma” package in the *R* software (version 3.6.3) was used to screened out DEGs between PAH samples and normal samples [14]. DEGs with a threshold of ∣log_2_ fold change (FC) | >1 and *P*_*adj*_ value <0.05 as the cut-off criteria were selected for further analyses [15, 16].

### 2.3. Functional Enrichment Analyses

The DAVID database (https://david.ncifcrf.gov/) is an online bioinformatic tool that provides a comprehensive set of functional annotation tools for researchers to understand the biological meaning behind a large number of genes [17, 18]. To further understand the function of DEGs in PAH, the DEGs were uploaded to the DAVID database to perform the GO enrichment analysis. The “clusterprofile” package in *R* software can perform statistical analysis and visualization of functional clustering of gene collections [19]. The KEGG pathway enrichment analysis of DEGs was performed using the “clusterprofiler” package [20]. Gene Ontology is a widely used ontology in the field of bioinformatics, which covers three aspects of biology: biological process (BP), cellular component (CC), and molecular function (MF) [21]. KEGG pathway annotation and analysis of DEGs can determine the major metabolic and signal transduction pathways involved in these genes [22]. The *P* value < 0.05 was regarded as the threshold values for remarkable enrichment.

### 2.4. Construction of the PPI Network

The Search Tool for the Retrieval of Interacting Genes (STRING) database (http://string-db.org/) is a database for searching known protein-protein interactions and predicting protein-protein interactions. It contains not only experimental data, data from text mining, but also results of protein-protein interactions predicted by using bioinformatic methods [23]. In this study, a PPI network of DEGs that was established with the minimum interaction score which was set as 0.4. Cytoscape is a very powerful tool for visualizing web data that can be used to demonstrate the interrelationships between a set of genes/proteins. Next, the PPI network was visualized by the Cytoscape software (version 3.7.2) [24]. CytoHubba from Cytoscape software was used to identify the hub genes in the PPI network. Five methods in Plug-in CytoHubba were used to select the hub genes in the PPI network, namely, edge percolated component (EPC), maximal clique centrality (MCC), maximal neighborhood component (MNC), node connect degree (degree), and node connect closeness (closeness). The genes scored in the top 10 by all 5 methods were selected as hub genes.

### 2.5. Prediction of miRNA and TF of Hub Genes and Construction miRNA-TF-Hub Gene Network

The online prediction tool microRNA Data Integration Portal (mirDIP) (http://ophid.utoronto.ca/mirDIP) is a web-based computational database that integrates dozens of bioinformatic tools used to predict the target miRNA of genes [25]. We used the mirDIP database to predict potential miRNA targeting of hub genes. The hub genes were submitted with the threshold set as follows: minimum score = very high, and the top five predicted miRNAs of every gene were chosen and listed. TRRUST (Transcriptional Regulatory Relationships Unraveled by Sentence-Based Text Mining) (version 2, http://www.grnpedia.org/trrust/) is an artificially annotated transcriptional regulatory network database [26]. The TRRUST database contains 800 human transcription factors (TFs) and 828 mouse TFs, with 8444 human and 6552 mouse TF-target regulatory relationships, respectively [26]. Then, the TRRUST database was used to predicted the TFs of the hub genes [27]. The TF-hub gene interaction pairs with *P* values <0.05 were selected to establish the regulatory network. Finally, the Cytoscape software was used to construct the miRNA-TF-hub gene network.

### 2.6. Drug-Gene/Protein Interactions

The Drug-Gene Interaction database (DGIdb) (http://dgidb.genome.wustl.edu/) was used to identify potential drug targets for the 5 identified hub genes. It contains data from 13 different sources on human drugs, drug-deliverable genes, and drug-gene interactions and currently contains more than fourteen thousand drug-gene interactions including more than six thousand drugs and two thousand human genes [28].

### 2.7. Diagnostic Significances of 5 Hub Genes

The evaluation of the diagnostic value of hub genes was analyzing by establishing a receiver operating characteristic (ROC) according to the hub gene expression data in 58 PAH patients and 25 normal control samples. The area under the curve (AUC) value of the ROC curve was used to determine the diagnostic effect of the hub genes in distinguishing patients with PAH from normal subjects. Usually, an AUC value of >0.85 indicated excellent diagnostic value [29, 30].

### 2.8. Statistical Analysis

The moderate *t*-test was used to screen out DEGs; Fisher's exact test to analysis was used to performed function enrichment analysis including GO and KEGG analysis [31]. All statistical analyses were performed in *R* version 3.6.3 software.

## 3. Results

### 3.1. Identification of DEGs in PAH

**Figure 2:**
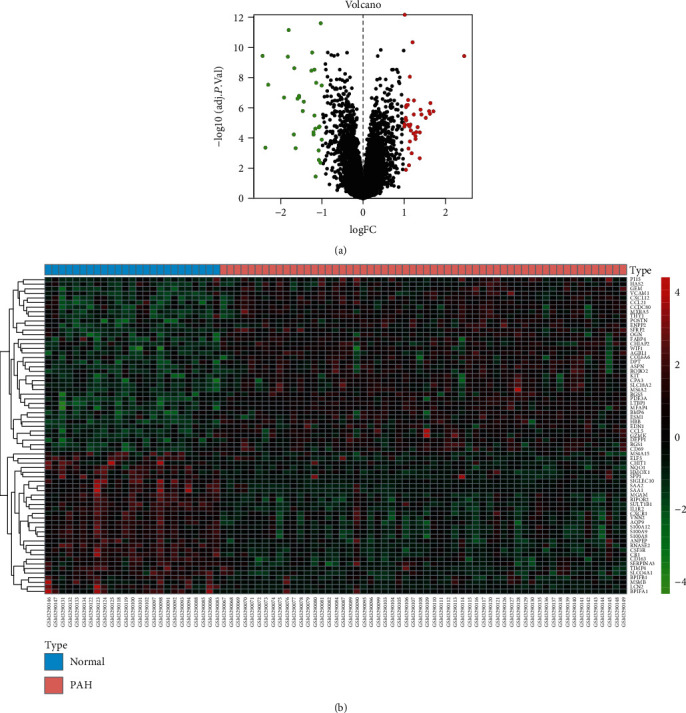
Identification of DEGs from GSE117261 dataset. (a) Volcano plot of GSE117261 via *R* software. Log FC: log2 fold change. (b) Heat map of differentially expressed gene expression. The heat map was generated using pheatmap package in *R*. The expression profiles greater than the mean are colored in red, and those below the mean are colored in green. Blue, normal lung tissues; orange, PAH specimens. PAH: pulmonary arterial hypertension.

To explore the drive genes of PAH, we first excavated the mRNA expression profiling (GSE117261) of PAH and normal tissue from GEO and filtrated DEGs compared to the normal tissue. In our result, 69 DEGs of PAH were obtained. Among them, 38 were upregulated expression (log_2_ FC > 1), and 31 were downregulated expression (log_2_ FC < −1) (Table 2 and Table S2). These DEGs were shown as a volcano plot and heat map in Figures 2(a) and 2(b).

### 3.2. Functional Enrichment Analyses

**Figure 3:**
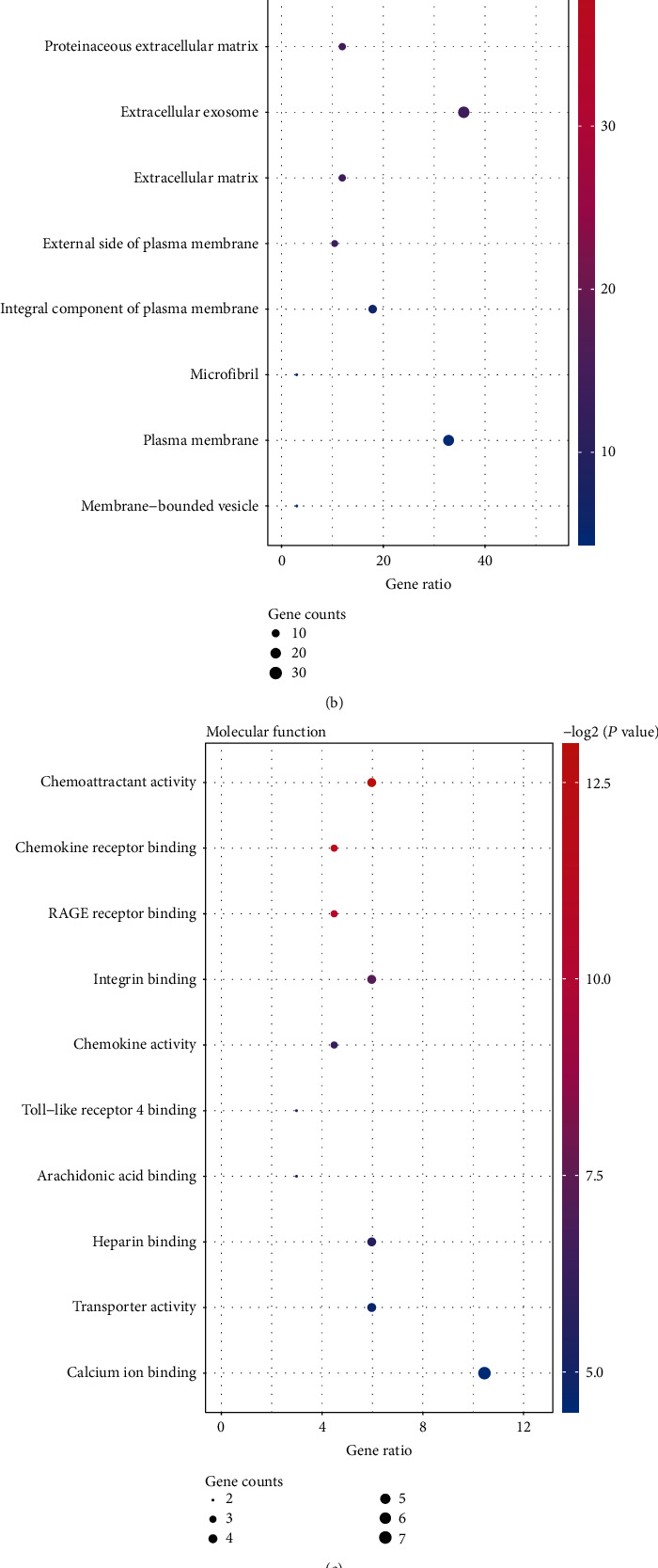
Top 10 enriched GO terms and top 10 KEGG pathways for differentially expressed genes. (a)–(c) GO term enrichment analysis for (a) biological process, (b) molecular function, and (c) cellular component. (d) KEGG pathway analysis. Node size represents gene ratio; node color represents *P* value. GO: Gene Ontology; KEGG: Kyoto Encyclopedia of Genes and Genomes.

To further understand the function of 69 DEGs in PAH, these 69 DEGs were uploaded to the DAVID database to perform the GO enrichment analysis (Table S3 and Figures 3(a)–3(c)). The *P* value <0.05 was regarded as the threshold values for remarkable enrichment. The top five GO terms of DEGs based on the *P* value are shown in Table 3. In BP analysis, it was shown that the DEGs were mainly involved in the inflammatory response, neutrophil chemotaxis, cellular response to tumor necrosis factor, immune response, and cell chemotaxis. In CC analysis, it was significantly involved in the extracellular region, extracellular space, proteinaceous extracellular matrix, extracellular exosome, and extracellular matrix. In addition, MF analysis showed that DEGs were mainly involved in chemoattractant activity, chemokine receptor binding, RAGE receptor binding, integrin binding, and chemokine activity. To obtain more information about the crucial pathways of these DEGs, a KEGG pathway analysis was performed (Table S4). The top ten KEGG terms of DEGs according to the *P* value are shown in Table 4 and Figure 3(d). KEGG enrichment analysis showed that DEGs were mainly involved in hematopoietic cell lineage, cytokine−cytokine receptor interaction, fluid shear stress and atherosclerosis, viral protein interaction with cytokine and cytokine receptor, and malaria (Table 4).

### 3.3. Construction of the PPI Network

**Figure 4:**
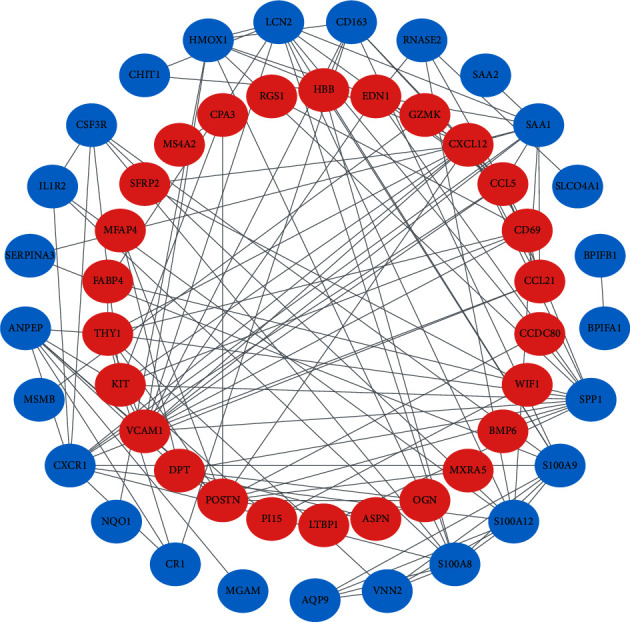
Construction of the PPI network. The nodes represent proteins, and the edges represent the interaction of proteins, while blue and red circles indicate downregulated and upregulated DEGs, respectively.

The PPI network of DEGs was constructed by using the STRING database and Cytoscape software. After removing the unconnected nodes, a PPI network that contained 51 nodes and 132 edges was constructed (Figure 4). The cytoHubba in the Cytoscape software was used to identify the hub genes of PAH scored in the top 10 by all 5 methods. These genes were SPP1, CXCL12, CXCR1, VCAM1, and CCL5, which may play an important role in PAH progression.

### 3.4. Prediction of miRNA and TF of Hub Genes and Construction miRNA-TF-Hub Gene Network

**Figure 5:**
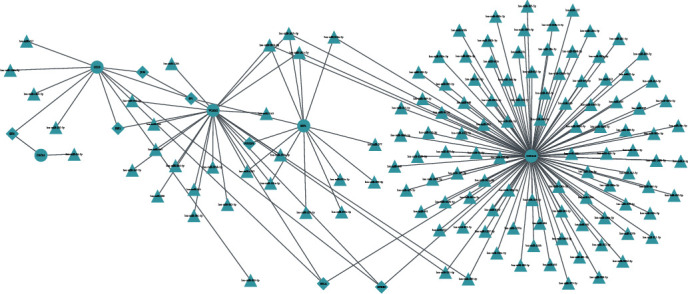
Construction of the miRNA-TF-hub gene interaction network. The circles represent hub genes, the triangles represent miRNAs, and the diamond represent TFs, respectively.

To further explore the mechanism of the hub genes, we investigated the potential interaction network of these hub genes. According to the mirDIP database and TRRUST database, there were 131 potential miRNAs (Table S5) and 7 TFs by targeting hub genes, and a miRNA-TF-hub gene interaction network was established. Finally, the miRNA-TF-hub gene interaction network was visualized via Cytoscape software (Figure 5).

### 3.5. Drug-Gene/Protein Interactions

According to the DGIdb database results, 17 drugs approved by the Food and Drug Administration (FDA) were screened out, including 4 drugs interacted with gene SPP1 (secreted phosphoprotein 1), 5 drugs with VCAM1 (vascular cell adhesion molecule 1), 6 drugs with CXCL12 (C-X-C motif chemokine ligand 12), and 2 drugs with CXCR1 (C-X-C motif chemokine receptor 1) (Table 5). Only one hub gene (CCL5) however does not have a direct drug target.

### 3.6. Diagnostic Significances of Hub Genes

**Figure 6:**
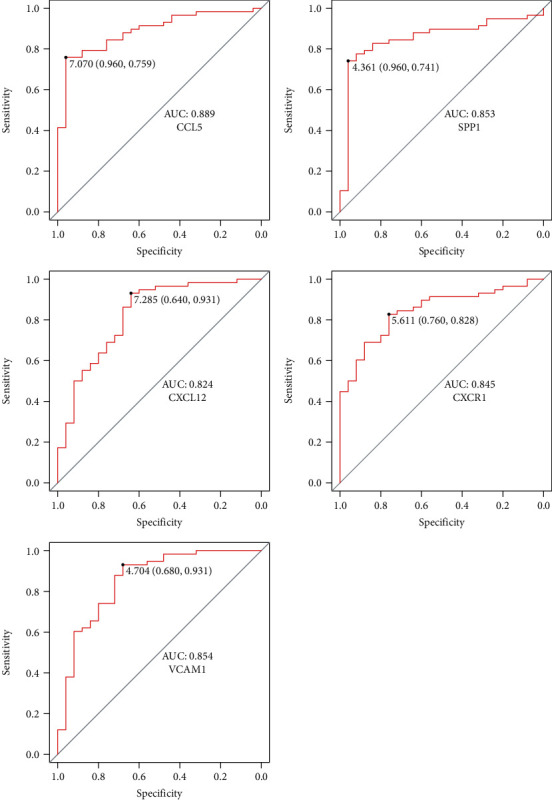
The ROC curve was used to evaluate the diagnostic value of hub genes.

The ROC curve was used to evaluate the diagnostic value of hub genes. The diagnostic value of hub genes in recognizing PAH tissues from normal control presented excellent diagnostic value with AUC of 0.889 (95% confidence interval (CI): 81.73%-96.07%), sensitivity of 75.9%, and specificity of 96.0% in CCL5; AUC of 0.854 (95% CI: 75.81%-95.08%), sensitivity of 93.1%, and specificity of 68.0% in VCAM1; and AUC of 0.853 (95% CI: 76.01%-94.61%), sensitivity of 74.1%, and specificity of 96.0% in SPP1 (Figure 6). However, CXCL12 and CXCR1 presented AUC of <0.85 (Figure 6).

## 4. Discussion

PAH still is a severe disease which is difficult to diagnosed and continuously makes patients and social suffering. The rapid development of high-throughput microarray technology and bioinformatic could provide more references for finding diagnostic biomarkers and prognostic suggestions for diseases.

In our work, GSE117261, a mRNA expression profile which downloaded from the NCBI-GEO database, was used to explore potential biomarkers and molecular mechanisms of PAH. Firstly, 69 DEGs (38 upregulated genes and 31 downregulated genes) between PAH lung tissues and normal lung tissues were screened out. Then, GO and KEGG analysis was conducted to gain more insights into the function of these DEGs in PAH. Further, we constructed a PPI network via the STRING database and Cytoscape software and screened out 5 hub genes (CCL5, CXCL12, VCAM1, CXCR1, and SPP1) from the PPI network. Moreover, the miRNA-hub gene interaction network was established. Finally, the candidate drug targeting hub genes with PAH were screened out via the DGIdb database.

CCL5 (C-C motif chemokine ligand 5), also known as SCYA5, RANTES, and TCP228, is one of the chemokine system genes and belongs to the C-C chemokine subfamily [32, 33]. CCL5 could be released from platelets, macrophages, fibroblasts, endothelium, and epithelial cells [34]. And it was reported that CCL5 plays multiple roles in the human tissue, including tissue repair/healing, fibrosis, angiogenesis, tissue and vascular remodeling, embryogenesis, and tumorigenesis [35–37]. The major contributors to promoting the development of PAH include but not limits to genetic factors, autoimmune, pulmonary vascular endothelium, and smooth muscle cell dysfunction. Nie and Tan et al. have reported that the CCL5-CCR5 pathway took part in macrophage recruitment and pulmonary vascular remodeling [38]. This evidence suggests that CCL5 might involve in the pathogenesis of PAH. Studies have shown that CCL5 is highly expressed in patients with PAH, and CCL5 gene knockout in mice could inhibit the development of Sugen5416/hypoxia-induced PAH [39, 40]. Changming et.al have reported that the overexpressed CCL5 was a risk factor for the pathogenesis of PAH, and CCL5 could exert vasoconstriction and remodel effects on the lung tissue in PAH [41]. In our study, we found that CCL5 is overexpressed in PAH patients, and this is consistent with previous research. What role does CCL5 play in pulmonary hypertension? It is reported that there may be in the following aspects: CCL5 may play an indirect role in PAH by inducing endothelin-converting enzyme 1 and endothelin-1, and endothelin-1 is a powerful endothelin-derived factor with strong vasoconstriction effects [42]. CCL5 is one of the genes regulating the NF-*κ*B signaling pathway [40]. Activation of NF-*κ*B is a feature of many chronic inflammatory conditions such as asthma and chronic obstructive pulmonary disease [40, 43, 44]. In atherosclerosis, activation of NF-*κ*B can be seen in macrophages endothelial cells and vascular smooth muscle cells in atherosclerotic plaques [45]. Studies show that NF-*κ*B may play an important role in PAH via mediating the cytokine-induced release of endothelin-1 [46]. PAH animal models have demonstrated that proinflammatory cytokines and chemokines are involved in the development of wild liliine-induced pulmonary hypertension [47, 48]. Therefore, we speculate that the crosstalk between the CCL5 and NF-*κ*B pathway plays an important role in the development of PAH, doing further researches of which might make a great progress in PAH. However, we found in the DGIdb database that there are currently no FDA-approved drugs targeting CCL5. Therefore, the development of drugs targeting CCL5 may be beneficial for the treatment of PAH.

SPP1 (secretory phospho-protein1), also known as OPN or osteopontin, is a chemokine-rich matrix phosphoglycoprotein, which mostly exists in human body fluids, lungs, gastrointestinal tract, and other organs. Previous studies have found that the SPP1 gene is highly expressed in idiopathic pulmonary fibrosis (IPF), the occurrence, and metastasis of multiple tumors. High expression of SPP1 promotes the occurrence of lung fibers by regulating the expression of many genes, such as LEP and KCNJ5 [49]. Chronic hypoxia and repeated chronic airway inflammation will lead to lung tissue destruction, fibrosis, and then increase pulmonary vascular resistance and eventually induce exacerbating PAH. Pulmonary fibrosis is a common reason for the formation of PAH. The comprehensive analysis showed that the high expression of SPP1 should be a driver of PAH. It has been reported that SPP1 plays a role in PAH via enhancing pulmonary vascular smooth muscle cell (PVSMC) proliferation [50, 51]. The expression level of SPP1 was related to the severity of PAH [52, 53]. These results suggest that SPP1 may be a prognostic indicator and therapeutic target for PAH. Targeted SPP1 therapy might reverse the development of pulmonary fibrosis and prevent or delay the progression of pulmonary hypertension. Further study of the molecular mechanism of SPP1 in pulmonary hypertension will be beneficial to the majority of patients.

VCAM1 encodes adhesion molecules induced by proinflammatory cytokines, and it has been reported that VCAM1 is increased in systemic sclerosis complicated with PAH [54]. VCAM1 on endothelial cells is involved in leukocyte adhesion and activates intracellular calcium release and NADPH oxidase Nox2, further promoting leukocyte migration [55, 56]. It has been reported that the concentration of VCAM-1 only increases in PAH-CTD, but not in patients with IPAH, suggesting that VCAM1 may play a specific role in PAH subtypes and may be related to the degree of inflammation, vascular injury, and antiangiogenesis [57]. However, other studies have found that VCAM1 is increased in both patients and animal models of IPAH [58, 59]. Therefore, the expression pattern and role of VCAM1 in pulmonary hypertension need to be further studied.

CXCL12 belongs to the CXC subfamily of chemokines, and increased blood CXCL12 levels are associated with right ventricular dysfunction in patients with idiopathic pulmonary hypertension [60]. The expression level of CXCL12 is significantly increased in the pulmonary vascular endothelium and the endothelium of the vasa vasorum of larger pulmonary vessels removed from PAH patients when compared to normal control [61, 62]. The neutralization effect of CXCL12 can reduce the infiltration of pulmonary macrophages, so as to improve pulmonary hypertension in rats [60]. The promoter region of CXCL12 contains binding sites for several transcription factors, including NF-*κ*B [63]. CXCL12 may play an in PAH via the NF-*κ*B signaling pathway. It is reported that the CXCL12/CXCR4/CXCR7 axis plays a central role in PAH [64]. These results also suggest that CXCL12 may be a therapeutic target for PAH.

CXCR1, also known as IL8R1, encoded a protein that is a member of the G-protein-coupled receptor family [65]. This protein is a receptor for interleukin 8 (IL8) [65]. Bone morphogenetic protein receptor-II (BMPR-II) expressed in pulmonary artery endothelial cells, which play an anti-inflammatory role by regulating the release of proinflammatory cytokines and promote the barrier function by inhibiting the migration of white blood cells to the pulmonary vascular wall [66]. In mice with deficient expression of BMPR-II, the decreased barrier function and the resulting PAH are the results of increased leukocyte recruitment caused by CXCR1/2 increase and inhibiting CXCR1/2 could be slow down to the progress of PAH [66]. These results suggest that CXCR1 may be a therapeutic target for PAH.

However, there are some limitations in this study. First, the sample size was relatively small. Second, this study was based on bioinformatics, and the results were based on computer analysis; so, the results need to be verified by experiments in vivo and in vitro.

## 5. Conclusions

Through bioinformatic analysis of microarray data sets, 5 hub genes (CCL5, CXCL12, VCAM1, CXCR1, and SPP1) were identified from DEGs between control samples and PAH samples. Studies showed that the five hub genes might play an important role in the development of PAH. These 5 hub genes might be potential biomarkers for the treatment of PAH. In addition, our work also indicated that paying more attention on studies based on these 5 hub genes might help to understand the molecular mechanism of the development of PAH.

## Figures and Tables

**Table 1:** Details of GEO dataset.

Dataset	Tissue	Platform	PAH	Normal	Reference (PMID)
GSE117261	Lung	GPL6244	58	25	30562042

Note: GEO: Gene Expression Omnibus; PAH: pulmonary arterial hypertension.

**Table 2:** Screening DEGs in PAH by integrated analysis of microarray.

DEGs	Gene names
Upregulated	LTBP1, PDE3A, HBB, MFAP4, BMP6, GEM, SFRP2, CCL5, SLC18A2, RGS5, DEPP, WIF1, ASPN, VCAM1, AGBL1, RGS1, CXCL12, POSTN, MS4A, MXRA5, DPT, OGN, CD69, CCL21, KIT, ENPP2, CPA3, THY1, EDN1, ESM1, GZMK, FABP4, ROBO2, COL6A6, CCDC80, CHIAP2, PI15, HAS2
Downregulated	CSF3R, RNASE2, SULT1B1, SAA2, S100A9, MGAM, NQO1, CR1, SIGLEC10, IL1R2, RIPOR2, S100A8, AQP9, S100A12, SAA1, CD163, LCN2, TIMP4, ANPEP, CXCR1, VNN2, HMOX1, BPIFA1, SLCO4A1, ELF5, BPIFB1, SPP1, SERPINA3, CHIT1, MS4A15, MSMB

DEGs: differentially expressed genes; PAH: pulmonary arterial hypertension.

**Table 3:** GO analysis of DEGs in PAH.

Category	Term	Count	*P* value	FDR
BP	Inflammatory response	12	1.21*E*-07	3.52*E*-05
BP	Neutrophil chemotaxis	7	1.51*E*-07	3.52*E*-05
BP	Cellular response to tumor necrosis factor	8	1.56*E*-07	3.52*E*-05
BP	Immune response	11	2.96*E*-06	5.01*E*-04
BP	Cell chemotaxis	6	4.06*E*-06	5.49*E*-04
CC	Extracellular region	30	2.33*E*-14	1.91*E*-12
CC	Extracellular space	27	1.84*E*-13	7.53*E*-12
CC	Proteinaceous extracellular matrix	8	4.62*E*-05	1.22*E*-03
CC	Extracellular exosome	24	5.97*E*-05	1.22*E*-03
CC	Extracellular matrix	8	8.64*E*-05	1.42*E*-03
MF	Chemoattractant activity	4	1.23*E*-04	2.11*E*-02
MF	Chemokine receptor binding	3	3.55*E*-04	3.03*E*-02
MF	RAGE receptor binding	3	6.92*E*-04	3.94*E*-02
MF	Integrin binding	4	6.48*E*-03	2.77*E*-01
MF	Chemokine activity	3	1.35*E*-02	4.10*E*-01

Note: GO: Gene Ontology; DEGs: differentially expressed genes; PAH: pulmonary arterial hypertension; BP: biological process; CC: cellular component; MF: molecule function; FDR: false discovery rate.

**Table 4:** KEGG enrichment analysis of DEGs in PAH.

Category	Term	Count	*P* value	FDR
hsa04640	Hematopoietic cell lineage	5	6.98*E*-05	6.63*E*-03
hsa04060	Cytokine-cytokine receptor interaction	7	2.71*E*-04	1.09*E*-02
hsa05418	Fluid shear stress and atherosclerosis	5	3.45*E*-04	1.09*E*-02
hsa04061	Viral protein interaction with cytokine and cytokine receptor	4	9.62*E*-04	2.29*E*-02
hsa05144	Malaria	3	1.38*E*-03	2.62*E*-02
hsa04614	Renin-angiotensin system	2	4.61*E*-03	7.30*E*-02
hsa04657	IL-17 signaling pathway	3	8.26*E*-03	1.10*E*-01
hsa04062	Chemokine signaling pathway	4	1.01*E*-02	1.10*E*-01
hsa04064	NF-kappa B signaling pathway	3	1.09*E*-02	1.10*E*-01
hsa05143	African trypanosomiasis	2	1.17*E*-02	1.10*E*-01

Note: KEGG: Kyoto Encyclopedia of Genes and Genomes; DEGs: differentially expressed genes; PAH: pulmonary arterial hypertension; FDR: false discovery rate.

**Table 5:** Candidate drug targeting genes with PAH.

Gene	Drug	Sources	Reference (PMID)
SPP1	Alteplase	NCI	12009309
SPP1	Gentamicin	NCI	11274264
SPP1	Tacrolimus	NCI	16103732
SPP1	Calcitonin	NCI	8013390
VCAM1	Cyclosporine	NCI	7694584
VCAM1	Carvedilol	DrugBank	17139284, 15374848, 17016423
VCAM1	Alcohol	DrugBank	18165316
VCAM1	Mercaptopurine	NCI	7694584
VCAM1	Dexamethasone	NCI	7694584
CXCL12	Vincristine	PharmGKB	27173875
CXCL12	Alemtuzumab	PharmGKB	27173875
CXCL12	Prednisone	PharmGKB	27173875
CXCL12	Cyclophosphamide	PharmGKB	27173875
CXCL12	Chlorambucil	PharmGKB	27173875
CXCL12	Rituximab	PharmGKB	27173875
CXCR1	Ibuprofen	TTD	N/A
CXCR1	Ketoprofen	DrugBank	9093816, 15974585, 11331079

Note: PAH: pulmonary arterial hypertension; NCI: National Cancer Institute; TTD: Therapeutic Target Database; N/A: not available.

## Data Availability

The data used to support the findings of this study are available from the corresponding author upon request.

## Supplementary Materials

Supplementary 1 Table S1. The demo data of the samples in GSE117261.

Supplementary 2 Table S2. The full length differential gene expression (DEGs) table with probe id, logFC, P value, adj.P val, t value, and gene name.

Supplementary 3 Table S3. GO analysis of DEGs.

Supplementary 4 Table S4. KEGG analysis of DEGs.

Supplementary 5 Table S5. Prediction potential miRNA targeting of hub genes.
