# Supplementary material for: Screening of Hub Genes Associated with Pulmonary Arterial Hypertension by Integrated Bioinformatic Analysis
Source: Biomed Res Int. 2021 Mar 22;2021:6626094. doi: 10.1155/2021/6626094 (PMC8010527; doi:10.1155/2021/6626094)
Supplement: Supplementary 1 — Table S1. The demo data of the samples in GSE117261. [file 6626094.f1.docx]

**Table S1.** The demo data of the samples in GSE117261.

|  | Control | PAH |
| --- | --- | --- |
| Samples, n | 25 | 58 |
| Age range, yr | 1-64 | 7-79 |
| Sex |  |  |
| Male | 18 | 15 |
| Female | 7 | 43 |
| PAH subtypes |  |  |
| IPAH |  | 31 |
| APAH |  | 18 |
| HPAH |  | 5 |
| Other |  | 4 |

Note: APAH = associated pulmonary arterial hypertension; HPAH = hereditary pulmonary arterial hypertension; IPAH = idiopathic pulmonary arterial hypertension.
